# Whole Genome Sequencing and Molecular Analysis of Carbapenemase-Producing *Escherichia coli* from Intestinal Carriage in Elderly Inpatients

**DOI:** 10.3390/microorganisms10081561

**Published:** 2022-08-03

**Authors:** Maria Giufrè, Giulia Errico, Monica Monaco, Maria Del Grosso, Michela Sabbatucci, Annalisa Pantosti, Marina Cerquetti, Michela Pagnotta, Manuela Marra, Maria Carollo, Angelo Rossini, Elena Fogato, Elisabetta Cesana, Flaminia Gentiloni Silverj, Dorjan Zabzuni, Marco Tinelli

**Affiliations:** 1 Department of Infectious Diseases, Istituto Superiore di Sanità, 00161 Rome, Italy; giulia.errico@iss.it (G.E.); monica.monaco@iss.it (M.M.); maria.delgrosso@iss.it (M.D.G.); michela.sabbatucci@iss.it (M.S.); annalisa.pantosti@iss.it (A.P.); marina.cerquetti@iss.it (M.C.); michela.pagnotta@guest.iss.it (M.P.); 2 Ministry of Health, Directorate General Health Prevention, Communicable Diseases and International Prophylaxis, 00144 Rome, Italy; 3 Core Facilities Technical-Scientific Service (FAST), Istituto Superiore di Sanità, 00161 Rome, Italy; manuela.marra@iss.it (M.M.); maria.carollo@iss.it (M.C.); 4 IRCCS Fondazione Santa Lucia, 00179 Rome, Italy; a.rossini@hsantalucia.it; 5 Golgi-Redaelli Geriatric Institute, 20146 Milan, Italy; e.fogato@golgiredaelli.it; 6 IRCCS Istituto Auxologico Italiano, San Luca Hospital, 20149 Milan, Italy; elisabetta.cesana@auxologico.it (E.C.); dorianzabzuni@gmail.com (D.Z.); martin.49@virgilio.it (M.T.); 7 IRCCS Fondazione Ca’ Granda Ospedale Maggiore Policlinico, 20122 Milan, Italy; flaminia.gentiloni@policlinico.mi.it; 8 Italian Society of Infectious and Tropical Diseases (SIMIT), 59100 Prato, Italy

**Keywords:** multidrug-resistance, high-risk *Escherichia coli* clones, carbapenemase, MLST, whole-genome sequencing, colonization, elderly

## Abstract

The spread of carbapenemase-producing (CP) Enterobacterales is currently a worldwide concern, especially in the elderly. Twelve CP-*E. coli* isolated from rectal swabs of colonized inpatients aged ≥65 years from four hospitals in two Italian cities (Milan and Rome) were analyzed by whole genome sequencing (WGS) to obtain multi-locus sequence typing (MLST), identification of carbapenemase-encoding genes, resistome, plasmid content, and virulence genes. MLST analysis showed the presence of 10 unrelated lineages: ST410 (three isolates from three different hospitals in two cities) and ST12, ST38, ST69, ST95, ST131, ST189, ST648, ST1288, and ST1598 (one isolate each). Most isolates (9/12, 75%) contained a serine-β-lactamase gene (5 *bla*_KPC-3_, 2 *bla*_KPC-2_, and 2 *bla*_OXA-181_), while three isolates harbored a metallo-β-lactamase gene (two *bla*_NDM-5_ and one *bla*_VIM-1_). In most CP-*E. coli*, the presence of more than one plasmid was observed, with the predominance of IncF. Several virulence genes were detected. All isolates contained genes enhancing the bacterial fitness, such as *gad* and *ter*C, and all isolates but one, *fim*H, encoding type 1 fimbriae. In conclusion, CP-*E. coli* clones colonizing elderly patients showed heterogeneous genetic backgrounds. We recommend strict surveillance to monitor and prevent the spread of successful, high-risk clones in healthcare settings.

## 1. Introduction

Antimicrobial resistance is currently one of the most worrying global public health problems. The European Centre for Disease Prevention and Control (ECDC) estimated the occurrence of almost 700,000 infections with antibiotic-resistant bacteria that accounted for nearly 33,000 attributable deaths each year in the EU/EAA countries [1]. The emergence and spread of carbapenem-resistant Enterobacterales (CRE) represents a major threat worldwide, especially in the most vulnerable patients, the elderly [2,3]. CRE infections, in particular those caused by carbapenemase-producing Enterobacterales (CPE), are associated with high mortality rates [4]. In Europe, CRE have been reported in most countries, although with very different prevalence. Most countries have a low overall prevalence, while few countries (such as Italy and Greece) report a high level of CRE endemicity [5]. Although the prevalence of invasive carbapenem resistant (CR)-*E. coli* is currently much lower than that of CR-*Klebsiella pneumoniae*, a small but significant increase in CR-*E. coli* in the EU/EEA population-weighted mean was reported between 2016 and 2020 (from 0.1% to 0.2%, with a 2020 country range of 0.0–0.8%) [5]. In Italy, an endemic country for CR-*K. pneumoniae*, the percentage of CR-*E. coli* has slightly increased from 0.3% in 2016 to 0.5% in 2020, in line with the European trend [5]. The principal resistance mechanism to carbapenems is the production of the carbapenemase enzymes in CPE isolates. Genes coding for carbapenemases are frequently located on plasmids, which can enhance the transfer of resistance determinants between bacteria and also spread the resistance to other species [6]. To date, few antibiotics are active against CPE, including the new β-lactam/β-lactamase inhibitor combinations such as ceftazidime–avibactam, meropenem–vaborbactam, and imipenem–relebactam [7,8]. However, resistance to these new molecules is already being reported [9].

Several virulence factors (VFs) have been identified in *E. coli*. VFs are proteins encoded by genes located either on the chromosome or on plasmids, and are classified in four main classes, based on their function: colonization (adhesion and invasion), fitness (iron acquisition and motility), toxins, and effectors [10]. The presence of VFs in colonizing isolates is concerning, since they can make *E. coli* capable of causing extraintestinal diseases in humans.

Asymptomatic colonization in the intestinal tract with carbapenemase-producing (CP)-*E. coli*, especially in the elderly, can lead to infections that are difficult to treat [11,12]. The potential spread of CP-*E. coli* among elderly patients or among people living in long-term care facilities (LTCF) is a matter of concern, since these infections are characterized by increased morbidity and mortality. Therefore, infection control measures are needed to prevent them [13]. In a previous longitudinal study aimed at evaluating the persistence of CPE colonization in elderly patients discharged from hospitals, our group found that out of 137 patients with CPE colonization, 91% carried CP-*K. pneumoniae* and 8.8% CP-*E. coli*. Notably, 43.1% of the available patients were still colonized after 4 months, representing a serious threat for the possibility of spreading such pathogens in the community or in healthcare settings [14]. Since little is known about colonizing CP-*E. coli* clones, the present study aims to describe the microbiologic and genomic features of isolates collected in the framework of a wider Italian research project on CPE colonization and persistence in the elderly [14].

## 2. Materials and Methods

### 2.1. Bacterial Strain Identification and Antimicrobial Susceptibility Testing (AST) 

CP-*E. coli* isolates were collected during the period January 2018 January 2020 from intestinal samples of hospitalized patients aged ≥65 years, within 3 days from hospital discharge in 4 hospitals located in two Italian cities (Milan and Rome, Italy), and during a 12-month follow-up, as described previously [14].

Samples processing, microbial identification, and antibiotic susceptibility testing were performed by the microbiology laboratories of the 4 hospitals, according to a shared protocol. Briefly, rectal swabs were collected in Amies medium (Copan, Brescia, Italy), refrigerated at 4 °C, and analyzed within 18 h. To detect CP-*E. coli*, swabs were streaked onto chromID CARBA SMART agar plates (bioMerieux, Marcy l’Etoile, France), and incubated at 35 °C for 48 h. Identification to species level was performed by a matrix-assisted laser desorption/ionization time-of-flight mass spectrometry (MALDI-TOF/MS) using a Microflex Biotyper^®^ LT (Bruker Daltonik GmbH, Bremen, Germany). Carbapenemase production was investigated using the agar tablet/disc diffusion method (KPC/MBL and OXA-48 Confirm Kit; ROSCO Diagnostica A/S, Taastrup, Denmark). Twelve confirmed CP-*E. coli* isolates were sent to the reference laboratory at Istituto Superiore di Sanità where further phenotypic and molecular characterizations were carried out. Antibiotic susceptibility testing (AST) was carried out by the reference broth microdilution method using lyophilized, custom microtitration plates (Merlin Diagnostika, Berlin, Germany), and by reference agar dilution for fosfomycin using an AD Fosfomycin panel (Liofilchem, Roseto degli Abruzzi, Italy). *E. coli* ATCC25922 was used as the control strain for AST. The interpretative breakpoints were based on EUCAST, version 11.0 [15].

### 2.2. Whole Genome Sequencing and In Silico Analysis

Genomic DNAs were extracted from an overnight culture using the NucleoSpin DNA extract kit (Macherey-Nagel, Duren, Germany). Whole-genome sequencing (WGS) was performed using Ion Torrent technologies (Life Technologies, Thermo Fisher Scientific, Waltham, MA, USA), according to the manufacturers’ instructions. De novo assembly of sequence reads was performed using SPAdes 3.9 software through the ARIES Galaxy server (https://w3.iss.it/site/aries/, accessed on 4 May 2022). MLST analysis was carried out according to the *E. coli* MLST website scheme (https://enterobase.warwick.ac.uk/species/index/ecoli, accessed on 4 May 2022). A core genome single-nucleotide polymorphism (SNPs) phylogeny was generated by CSI Phylogeny at the Center for Genomic Epidemiology (https://cge.cbs.dtu.dk/services/, accessed on 4 May 2022) using the complete genome of *E. coli* strain K12 (accession no. NC_000913.2) as a reference. The resulting maximum likelihood phylogenetic tree was visualized by uploading the Newick file to the Interactive Tree of Life platform, iTOL (http://itol.embl.de/upload.cgi, accessed on 4 May 2022) [16]. In silico analysis of the assembled contigs were performed using tools available at the CGE server SeroTypeFinder and FimTyper were used to predict serotype and Fim type isolates, respectively. Contigs were also screened for plasmid and resistance gene content using PlasmidFinder and ResFinder tools, respectively, at the CGE server. Plasmids of the IncF, IncH1, IncH2, IncI1, IncN, or IncA/C types were subtyped by assigning a replicon allele at the plasmid MLST site (https://pubmlst.org/plasmid/, accessed on 4 May 2022). Identification of virulence genes related to CP-*E. coli* was performed by VirulenceFinder at CGE.

## 3. Results

### 3.1. Antimicrobial Susceptibility Testing and Characterization of Resistance Genes

Between 2018 and 2020, 12 CP-*E. coli* isolates were recovered from elderly inpatients and were available for antibiotic susceptibility testing and WGS. 

**Figure 1:**
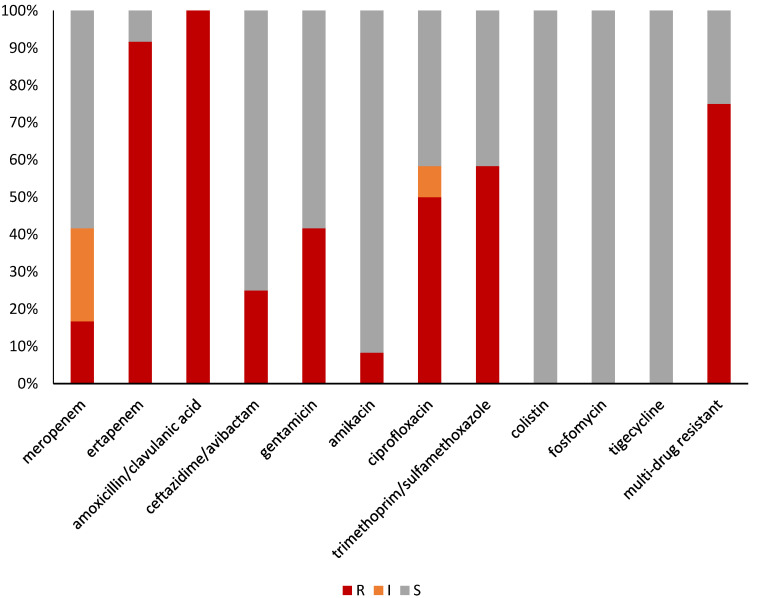
Antimicrobial susceptibility profiles of 12 CP-*E. coli* isolates from intestinal carriage in elderly patients. Red/orange/grey color in each column indicates percentage of resistant/susceptible, increased exposure/susceptible isolates, respectively.

Antibiotic susceptibility profiles of CP-*E. coli* isolates are shown in Figure 1. All isolates were resistant to at least one of the tested carbapenem (meropenem or ertapenem). The resistance rates to meropenem or ertapenem were 16.7% (2/12) and 91.7% (11/12), respectively. Seven isolates (58.3%) were resistant to trimethoprim/sulfamethoxazole, six (50.0%) to ciprofloxacin, and five (41.7%) to gentamicin. Three isolates (25.0%) were resistant to ceftazidime/avibactam and one (8.3%) to amikacin. All isolates were susceptible to colistin, fosfomycin, and tigecycline. 

### 3.2. WGS Analysis of CP-E. coli

Whole-genome sequencing and in silico analysis was carried out on all the CP-*E. coli* isolates. Serotype O8:H9 was relatively the most frequent (3/12) and found in the isolates belonging to the same ST410; the remaining nine isolates were classified into nine different serotypes (Table 1). MLST analysis showed the presence of 10 CP-*E. coli* lineages: ST410 (3 isolates), ST12, ST38, ST69, ST95, ST131, ST189, ST648, ST1288, and ST1598 (one isolate each) (Table 1). Six different *fim*H types were detected; *fim*H24 was relatively the most frequent (3/12) and found in isolates belonging to two different STs (ST410 and ST38).

**Figure 2:**
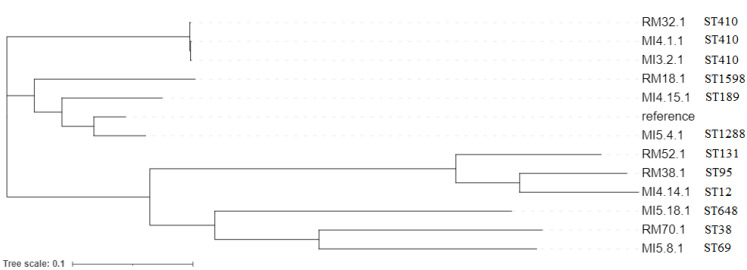
SNP-based phylogeny of CP-*E. coli* isolates from intestinal carriage in elderly patients. The maximum likelihood tree was rooted in a reference isolate *E. coli* strain K12 (accession no. NC_000913.2). Sequence type (ST) is shown for each isolate.

A core genome SNP Tree showed that the isolates were not closely related, as no major clades were present (Figure 2), except the branch including the three ST410 isolates. No single-locus variants were detected, and all STs belonged to different Clonal Complexes (CCs), except ST1288 and ST1598, which did not cluster in any established CC. The three isolates belonging to ST410 did not represent an epidemic cluster, since they originated from three different hospitals in the two cities: two isolates from the same city differed by 21 SNPs, while the third isolate differed by 59 and 60 SNPs from the other two, respectively (Supplementary Table S1).

### 3.3. Plasmid Content of CP-E. coli

Plasmid content was determined by PlasmidFinder and is summarized in Table 1. All CP-*E. coli* isolates harbored plasmids, and in all but one, the simultaneous presence of more than one plasmid was detected. Overall, IncF was the predominant plasmid found in most of the CP-*E. coli* (10/12, 83.3%), followed by Col (6/12, 50%). Ten isolates carried IncFIB and IncFII plasmids; IncFIA was present in five isolates. Besides IncF, several plasmid classes (IncA, IncB/O/K/Z, IncI-1, IncQ, IncR, IncX1, IncX3, and IncY), were found among the isolates in different combinations. Isolates belonging to ST410 shared the same pMLST for IncF [F1;A1;B49]. The remaining isolates had different pMLST.

Plasmid location of carbapenemase-encoding genes was identified in seven cases due to the short read technology used. *bla*_KPC_ genes were located on an IncFII(K) plasmid in four cases and on an IncX3 in one case, while *bla*_OXA-181_ were on a ColKP3 plasmid in two cases.

### 3.4. Resistome of CP-E. coli

The resistome of the isolates is shown in Table 1. Characterization of the genes responsible for carbapenemase production showed that most isolates (7/12, 58.3%) harbored *bla*_KPC_ genes (five isolates *bla*_KPC-3_ and two *bla*_KPC-2_) (Table 1). Among the remaining isolates, two (16.7%) were positive for *bla*_NDM-5_, two (16.7%) for *bla*_OXA-181_, and one (8.3%) for *bla*_VIM-1_. No isolate co-harbored different carbapenemase-encoding genes.

Extended-spectrum β-lactamase (ESBL) genes were found in nearly all isolates (10/12 CP-*E. coli*, 83.3%). In particular, *bla*_CTX-M-15_ was found in six isolates, *bla*_CMY-2_ in three isolates, and *bla*_SHV-12_ in one isolate. ESBL genes were associated with different carbapenemase genes (Table 1). Aminoglycoside-modifying enzymes were identified in 10/12 isolates (83.3%): five isolates carried *aac(6′)-Ib-cr*, associated with *aadA5*, *aadA2,* or *aadA1*. Plasmid-mediated resistance to fluoroquinolones was found in six isolates, five carrying *aac(6′)-Ib-cr*, alone or in combination to *qnrS1*, and one carrying only *qnrS1*. Sulfamethoxazole/trimethoprim resistance genes *dfrA12*, *dfrA14*, and/or *dfrA17* were found in eight isolates. The macrolide resistance *mph(A)* gene was found in six isolates; sulphonamide resistance *sul1* and *sul2* genes were detected in six and seven isolates, respectively. Tetracycline resistance genes *tet(A)*, *tet(B)*, and *tet(D)* were detected in seven isolates. Chloramphenicol resistance genes *catB2* and *catB3* were found in three isolates. No streptomycin, colistin, or fosfomycin acquired resistance genes were found. Notably, the three ST410 isolates carried *bla*_NDM-5_ or *bla*_OXA-181_ genes, with different combinations of resistance genes and plasmids.

### 3.5. Virulence Genes of CP-E. coli

The virulence genes identified in CP-*E. coli* are summarized in Table 2.

Virulence genes were detected in each isolate with a variable distribution. Several virulence genes coding for virulence factors (VFs) involved in adhesion (83% of the isolates), capsule formation (50%), iron acquisition (67%), serum resistance (67%), and toxicity (25%) were detected. All isolates possessed the glutamate decarboxylase gene *gad*, essential in maintaining the bacterial physiological pH in acidic conditions, and *terC*, a metal sensing stress response system. The sequences encoding adhesion proteins (adhesins, fimbriae, pilin, and outer membrane proteins) were found in most strains: the *fim*H gene, coding for the type 1 fimbria, was found in eleven isolates; *lpfA*, encoding fimbriae, was found in six isolates; and *yfcV,* associated with *papA/C* and coding for pilin, was found in four isolates. *Kps* genes, encoding the group two capsule, were detected in six isolates. Several siderophores for iron acquisition were detected: *sitA*, coding for an iron transport protein, in seven isolates, and *chuA* and/or *fyuA* plus *irp2*, coding for iron receptors, in six and five isolates, respectively. Regarding serum resistance, the *traT* gene, coding for an outer membrane protein for complement resistance, was found in eight isolates. *iss*, for increased serum survival, and *ompT*, an outer membrane protease, were found in five isolates. Virulence genes coding for VFs related to toxicity were only found in 25% of isolates: *usp*, an uropathogenic specific protein, and *vat*, a vacuolating autotransporter toxin, were found in three and two isolates, respectively.

## 4. Discussion

The prevalence of multidrug-resistant (MDR) *E. coli* strains is increasing worldwide, mainly due to the spread of mobile genetic elements carrying resistance determinants such as ESBLs and carbapenemase-encoding genes. According to a recent survey on CPE in Europe (EuSCAPE), resistance to carbapenems in *E. coli* is uncommon, and only a small proportion of CP-*E. coli* isolates (0.2–0.5%) is present among European and Italian isolates [17,18]. In our previous studies, few *E. coli* isolates from infection cases were found to be resistant to carbapenems; some did not produce any carbapenemase, and others carried a bla_NDM-5_ or *bla*_VIM-1_ gene [19,20,21]. In a carriage study in the elderly, our group observed a 1% prevalence of intestinal CP-*E. coli* colonization [22]. CP-*E. coli* infections are difficult to treat because isolates are frequently resistant to several different antibiotic classes, and the therapeutic options are scarce.

In this study, we confirmed CP-*E. coli* to be resistant to several antibiotics besides carbapenems: the most frequent resistance phenotype was resistance to amoxicillin/clavulanic acid, trimethoprim-sulfamethoxazole, ciprofloxacin, and/or gentamicin, in line with data from the European Antimicrobial Resistance Surveillance Network [5,17]. However, all isolates maintained susceptibility to colistin, fosfomycin, and tigecycline. The most common mechanism of carbapenem resistance was KPC production that represents the main cause of the endemic CPE situation in Italy [18]. We also found that three isolates, all harboring a carbapenemase gene of the metallo β-lactamase, were resistant to ceftazidime/avibactam, as already reported [23].

As reported in the literature [24,25], a high genetic diversity was observed: by MLST genotyping, no specific clone was associated with the presence of a carbapenemase, as we found 10 different lineages in the 12 isolates tested. Specifically, we found clones that are frequently associated with urinary tract infections or bloodstream infections, such as ST131, ST69, ST410, and ST95. ST131 is the most frequent *E. coli* clone associated with fluoroquinolone resistance and ESBL production, predominating globally and causing most human infections [19,26]. Carbapenem-resistant ST131 isolates are not common, but few cases have been previously reported worldwide [27]. The finding of this pandemic and diffusible clone carrying a carbapenemase-encoding gene is particularly worrisome due to its high potential to cause severe infections. ST410 and ST648 are now emerging as other globally disseminated *E. coli* clones that can carry carbapenemases [28,29]. ST410 was the relatively more frequent strain detected in our study, confirming the emergence of such a strain as one of the main drivers of carbapenem resistance in *E. coli* that needs to be monitored [24,25,28]. The ST410 isolates were associated with different combinations of plasmid/resistance genes, even if their virulence gene content was similar, confirming that they are not part of an epidemic cluster. ST69 strains are often multidrug-resistant [30]; ST95 are characterized by a relatively low frequency of multidrug resistance but sometimes associated with *bla*_KPC_ [31].

Plasmid analysis showed that the IncF plasmid is present in several different STs examined in this study, as already reported [32]. Besides IncF, we found several plasmid classes, carrying several resistance genes belonging to different antimicrobial classes, reflecting the MDR genotypes of the CP-*E. coli* isolates [32]. Serine-β-lactamase genes were more frequently detected than metallo-β-lactamase genes (75% vs. 25%), reflecting the Italian epidemiology [18]. Acquired resistance genes were observed in all strains of the different STs. The majority of isolates carried carbapenemase in association with different ESBL and/or AmpC β-lactamase genes; *bla*_CTX-M-15_ and *bla*_OXA-9_ were the most frequent ESBL. *bla*_CTX-M-15_ has been reported as the most prevalent ESBL enzymes in our country in the last decade and has usually been associate with ExPEC *E. coli* isolates [19,22].

In this study, virulence genes coding for VFs such as adhesins and siderophores were present in almost all isolates, with an overall heterogeneity in their distribution, as being reported by other authors in pathogenic *E.coli* [33]. The presence of virulence genes is worrying because it enhances the ability of the microorganisms to survive in the colonized host and eventually cause disease. The majority of isolates contained genes for VFs involved in adhesion (mostly the fimbrial *fim*H gene), regarded as the first step in pathogenesis, enabling close contact of the bacteria with the host cell wall, which is essential for colonizing specific niches [34]. VFs of the siderophore systems related to iron acquisition and essential for bacterial growth, especially during bacterial infection [35], were found in 67% of the isolates.

Such a wide distribution of clones and resistance genes and/or virulence genes confirms that no particular CP-*E. coli* clone has emerged until now in colonization/infection, as found in other studies [36,37,38,39].

## 5. Conclusions

In conclusion, by analyzing genome sequences of CP-*E. coli* isolates from intestinal colonization of the elderly, we found several different clones carrying a multitude of resistance and virulence genes. The combination of multiple resistance and virulence genes may play a fundamental role in increasing the fitness and the potential to cause disease of CP-*E. coli*. This is concerning, since it could result in the spreading of these MDR strains in hospital settings or the community and in the development of difficult to treat infections. Continued monitoring of CP-*E. coli* will be critical to prevent the expansion of high-risk clones as well as to limit the dissemination of antimicrobial-resistance genes to fight the burden of AMR.

## Figures and Tables

**Table 1:** Molecular and resistome analysis of 12 CP-*Escherichia coli* isolates from carriage in elderly patients.

Isolate	Serotype	ST	CC	fimH	Plasmid Content	pMLST *	β-Lactamases	Aminoglycoside Modifying Enzimes	FQs	MLS	Phenicol	Sulphonamide	TET	Trimethoprim
MI3.2.1	O8:H9	410	ST23	24	IncFIA, IncFIB, IncFII, IncQ1	IncF[F1:A1:B49]	NDM-5, CTX-M-15, CMY-2, OXA-1, TEM-1B	*aac(6′)-Ib-cr*, *aadA2*, *aadA5*, *aph(6)-Id*, *aph(3″)-Ib*, *aac(3)-IId*	*aac(6′)-Ib-cr*	*mph(A)*	*catB3*	*sul1*, *sul2*	*tet(B)*	*dfrA12*, *dfrA17*
MI4.1.1	O8:H9	410	ST23	24	IncFIA, IncFIB, IncFII, Col (BS512)	IncF[F1:A1:B49]	NDM-5, CTX-M-15, CMY-2	*aac(6′)-Ib-cr*, *aadA2*, *aph(6)-Id*, *aph(3″)-Ib*, *aac(3)-IId*	*aac(6′)-Ib-cr*	*mph(A)*		*sul1*, *sul2*		*dfrA12*
RM32.1	O8:H9	410	ST23	21	IncFIA, IncFIB, IncFII, IncQ1, IncX3, IncY, Col (BS512), ColKP3	IncF[F1:A1:B49]	OXA-181, CTX-M-15CMY-2, OXA-1, TEM-1B	*aac(6′)-Ib-cr*, *aadA5*, *aph(6)-Id*, *aph(3″)-Ib*, *aac(3)-IId*	*aac(6′)-Ib-cr*, *qnrS1*	*mph(A)*	*catB3*	*sul1*, *sul2*	*tet(B)*	*dfrA17*
MI4.14.1	-:H5	12	ST12	5	IncFIA, IncFIB, IncFII, IncX1, IncB/O/K/Z, Col156, ColKP3	IncF[F1:A6:B20]	OXA-181, TEM-1B	*aadA5*, *aph(6)-Id*, *aph(3″)-Ib*, *aac(3)-IId*		*mph(A)*		*sul1*, *sul2*	*tet(A)*, *tet(C)*	*dfrA17*
MI4.15.1	O188:H21	189	ST165	54	IncX3		KPC-3, SHV-182	*aadA5*				*sul2*	*tet(A)*	*dfrA17*
MI5.4.1	O9:H9	1288	-	54	IncFIB, IncFII, ColpVC	IncF[K2:A-:B-]	KPC-2	*aph(3″)-Ib*, *aph(6)-Id*					*tet(B)*	
MI5.8.1	O15:H18	69	ST69	27	IncA, IncB/O/K/Z	IncA/C [12]	VIM-1, SHV-12	*aac(6′)-Ib-cr*, *aadA1*, *aph(6)-Id*, *aph(3″)-Ib*, *aac(6′)-Ib3*	*aac(6′)-Ib-cr*, *qnrS1*	*mph(A)*	*catB2*	*sul1*, *sul2*		*dfrA14*
MI5.18.1	-:H6	648	ST648	27	IncFIB, IncFII	IncF[K2:A-:B-]	KPC-2, CTX-M-15, OXA-9							
RM18.1	O9:H4	1598	-	-	IncFIB, IncFII, IncI1-I, IncR, IncX3, Col (BS512)	IncF[K2:A-:B53], IncI1[154]	KPC-3, CTX-M-15, OXA-9, SHV-182, TEM-1B	*aph(3″)-Ib*, *aph(6)-Id*	*qnrS1*			*sul2*	*tet(A)*	*dfrA14*
RM38.1	O1:H7	95	ST95	30	IncFIB, IncFII	IncF[F2:A-:B1]	KPC-3, OXA-9, TEM-1A							
RM52.1	O25:H4	131	ST131	30	IncFIA, IncFIB, IncFII	IncF[K2:A4:B1]	KPC-3, CTX-M-15, OXA-1, OXA-9, TEM-1A	*aac(6′)-Ib-cr*, *aadA5*, *aac(3)-IIa*	*aac(6′)-Ib-cr*	*mph(A)*	*catB3*	*sul1*	*tet(A)*	*dfrA17*
RM70.1	-:H9	38	ST38	24	IncFIB, IncFII	IncF[K2:A-:B-]	KPC-3, OXA-9, TEM-1A							

* Allele numbers assigned by pMLST are in bracket; ST-sequence type; CC-Clonal Complex; MLS- Macrolide, lincosamide, and streptogramin; FQs- Fluoroquinolones; TET-tetracycline.

**Table 2:** Virulence gene content, according to their functions, of 12 CP-*Escherichia coli* isolates from carriage in elderly patients.

Isolate	Adhesins	Capsule	Siderophores	Serum Resistance	Toxins	Other
MI3.2.1	*fimH*, *lpfA*					*gad*, *terC*
MI4.1.1	*fimH*, *lpfA*					*gad*, *terC*
RM32.1	*fimH*, *lpfA*					*gad*, *terC*
MI4.14.1	*fimH*, *focC*, *papA/C*, *sfaD*, *yfcV*	*kpsE/MII*	*chuA*, *fyuA*, *iroN*, *irp2*, *sitA*	*iss*, *ompT*, *traT*	*cea*, *senB*, *usp*, *vat*	*gad*, *terC*
MI4.15.1	*fimH*, *lpfA*					*gad*, *terC*
MI5.4.1	*fimH*		*sitA*	*traT*		*gad*, *terC*
MI5.8.1	*fimH*, *lpfA*	*kpsE/MII*	*chuA*, *fyuA*, *irp2*, *sitA*	*iss*, *ompT*, *traT*		*gad*, *terC*
MI5.18.1	*fimH*, *lpfA*, *papA/C*, *yfcV*	*kpsE/MII*, *neuC*	*chuA*, *fyuA*, *irp2*	*ompT*, *traT*		*gad*, *terC*
RM18.1			*sitA*	*iss*, *traT*		*gad*, *terC*
RM38.1	*fimH*, *papA/C*, *yfcV*	*kpsE/MII*, *neuC*	*chuA*, *fyuA*, *ireA*, *iroN*, *irp2*, *iucC*, *sitA*	*iss*, *ompT*, *traT*	*cia*, *hlyF*, *usp*, *vat*	*gad*, *terC*
RM52.1	*afaA/C/D*, *fimH*, *hra*, *iha*, *nfaE*, *papA/C*, *yfcV*	*kpsE/MII*	*chuA*, *fyuA*, *irp2*, *iucC*, *sitA*	*iss*, *ompT*, *traT*	*cnf1*, *sat*, *usp*	*gad*, *terC*
RM70.1	*fimH*, *hra*	*kpsE/MII*	*chuA*, *sitA*	*traT*		*gad*, *terC*

## Data Availability

Sequence data were submitted to GenBank at NCBI under the BioProject PRJNA789446.

## Supplementary Materials

The following supporting information can be downloaded at: https://www.mdpi.com/article/10.3390/microorganisms10081561/s1, Table S1: Matrix of core genome single nucleotide polymorphisms (SNPs) between *E. coli* isolates from the elderly.
