# Supplementary material for: Whole Genome Sequencing and Molecular Analysis of Carbapenemase-Producing Escherichia coli from Intestinal Carriage in Elderly Inpatients
Source: Microorganisms. 2022 Aug 3;10(8):1561. doi: 10.3390/microorganisms10081561 (PMC9413394; doi:10.3390/microorganisms10081561)
Supplement: Supplementary file 1 [file microorganisms-10-01561-s001.zip › microorganisms-1825240-supplementary.pdf]

Supplementary Table S1. Matrix of core genome single nucleotide polymorphisms (SNPs) between *E. coli* isolates from the elderly.

	MI3.2.1	MI4.1.1	MI4.14.1	MI4.15.1	MI5.18.1	MI5.4.1	MI5.8.1	reference	RM18.1	RM32.1	RM38.1	RM52.1	RM70.1
MI3.2.1	0	21	45394	21787	41026	20907	39304	20478	23720	60	44903	44829	39837
MI4.1.1	21	0	45402	21795	41039	20912	39316	20485	23727	59	44911	44837	39845
MI4.14.1	45394	45402	0	42987	41088	41491	46672	39480	44868	45401	16907	22569	46770
MI4.15.1	21787	21795	42987	0	38884	13517	37576	11920	19401	21790	42514	42505	38279
MI5.18.1	41026	41039	41088	38884	0	37698	36485	35792	40884	41036	40941	41500	35911
MI5.4.1	20907	20912	41491	13517	37698	0	35845	6773	17139	20909	41126	40990	37017
MI5.8.1	39304	39316	46672	37576	36485	35845	0	34372	39498	39315	46233	46936	28856
reference	20478	20485	39480	11920	35792	6773	34372	0	17829	20484	39184	39130	35198
RM18.1	23720	23727	44868	19401	40884	17139	39498	17829	0	23720	44332	44302	39995
RM32.1	60	59	45401	21790	41036	20909	39315	20484	23720	0	44910	44838	39848
RM38.1	44903	44911	16907	42514	40941	41126	46233	39184	44332	44910	0	21914	46614
RM52.1	44829	44837	22569	42505	41500	40990	46936	39130	44302	44838	21914	0	46916
RM70.1	39837	39845	46770	38279	35911	37017	28856	35198	39995	39848	46614	46916	0
